# High Precision Classification of Resting and Eating Behaviors of Cattle by Using a Collar-Fitted Triaxial Accelerometer Sensor

**DOI:** 10.3390/s22165961

**Published:** 2022-08-09

**Authors:** Kim Margarette Corpuz Nogoy, Sun-il Chon, Ji-hwan Park, Saraswathi Sivamani, Dong-Hoon Lee, Seong Ho Choi

**Affiliations:** 1 ThinkforBL Consultancy Services, Seoul 06236, Korea; 2 Department of Animal Science, Chungbuk National University, Cheongju City 28644, Korea; 3 Department of Biosystems Engineering, Chungbuk National University, Cheongju City 28644, Korea

**Keywords:** machine learning, cattle behavior, triaxial accelerometer, sampling rate, sampling interval

## Abstract

Cattle are less active than humans. Hence, it was hypothesized in this study that transmitting acceleration signals at a 1 min sampling interval to reduce storage load has the potential to improve the performance of motion sensors without affecting the precision of behavior classification. The behavior classification performance in terms of precision, sensitivity, and the F1-score of the 1 min serial datasets segmented in 3, 4, and 5 min window sizes based on nine algorithms were determined. The collar-fitted triaxial accelerometer sensor was attached on the right side of the neck of the two fattening Korean steers (age: 20 months) and the steers were observed for 6 h on day one, 10 h on day two, and 7 h on day three. The acceleration signals and visual observations were time synchronized and analyzed based on the objectives. The resting behavior was most correctly classified using the combination of a 4 min window size and the long short-term memory (LSTM) algorithm which resulted in 89% high precision, 81% high sensitivity, and 85% high F1-score. High classification performance (79% precision, 88% sensitivity, and 83% F1-score) was also obtained in classifying the eating behavior using the same classification method (4 min window size and an LSTM algorithm). The most poorly classified behavior was the active behavior. This study showed that the collar-fitted triaxial sensor measuring 1 min serial signals could be used as a tool for detecting the resting and eating behaviors of cattle in high precision by segmenting the acceleration signals in a 4 min window size and by using the LSTM classification algorithm.

## 1. Introduction

Research works about cattle behaviors reported that eating and ruminating behaviors are altered when diagnosed with metabolic diseases [1,2,3] or mastitis [4]. It was also reported that deviation of cattle from their normal behavior in terms of feeding affects milk production and reproduction, particularly in dairy cattle [3]. In addition, when dairy cattle are affected with metabolic diseases such as ketosis, the standing behavior of the animals is reduced [5] and lying behavior is increased [6]. Other activities of cattle such as reduced sleeping increased inflammatory responses of the animal [7] and the walking behavior, and the overall activity of cattle affected by lameness decreased [8]. Clearly, understanding the behaviors of the animals can lead to the advanced detection of health conditions of the cattle whether they are healthy or ill with the disease.

With the birth of artificial intelligence, agriculture in livestock production has seen large development. One of the many innovations is the use of smart computing technologies such as accelerometer sensors that collect and identify behavioral data. These sensors have aided livestock farmers in monitoring the health, feeding, and production of dairy cows and beef cattle. The approach of these accelerometer sensors focuses on the well-being and the performance of the individual animal rather than the traditional herd-based monitoring, thus, providing the specific needs of the livestock animals more accurately. The monitoring systems can collect data 24 h/day over extended periods since these accelerometer sensors are typically attached directly to the animals. These on-body-based accelerometer sensors can not only reduce the physical tasks of the farmers, but also allow researchers and farmers to accurately recognize the health of the animals by reducing bias related to interpreting behaviors from the monitored movements of the animals. Commonly used sensors to quantify movements are inertial measurement units (IMU), gyroscopes, and triaxial accelerometers. The IMUs are used for devices that require the exact position such as robotic arms and exoskeletons [9], while gyroscopes are used for monitoring the orientation of a moving subject such as swimming accelerations of sea lions [10], and triaxial accelerometers are used to measure the linear acceleration dynamic of the subject such as monitoring ingestive movements of cattle [11]. In measuring the vector sum of acceleration due to gravity and the movement of the animals, accelerometer sensors could be the most suitable tool. Triaxial accelerometers were used to detect posture and overall behavior [12], drinking behavior [13], feed intake [14], and grazing-related activities [15] of the cattle. In general, these accelerometer sensors normally measure the behaviors of the animals using short window sizes at 3 s, 5 s, and 10 s [16,17], or medium window sizes of 20 s and 30 s [18], and semi-long window sizes of 32 s, 64 s, and 96 s [19]. Measurement of the acceleration signals at very short sampling intervals or fractions of a second and analysis at short window sizes, however, is impractical in terms of its large data load that shortens the battery life of the sensors. In this study, the aim was to focus on the classification of cattle behavior in three main categories. The first main behavior was the active behavior which includes standing and walking. Standing and walking are considered minor daily activities of cattle because the animals do not spend much time on them. The second main behavior was the eating behavior which includes the routine feeding and ruminating activities of the cattle. Feeding composed of chewing is a short-durational movement while ruminating composed of movements in the jaw and in the body is considered a long-duration activity. Finally, the resting behavior which includes lying and sleeping activities of the cattle is defined as durational activity. Based on the mid- to long-duration time characteristics of the movements of the three main behaviors of the cattle, it was hypothesized that the sampling interval of 1 min of the triaxial accelerometer will recognize the activities of the animals at high precision and accuracy.

In general, the ultimate objective of the study is to minimize the number of calculations and possibly reduce the power consumption of the sensors by using a triaxial accelerometer that measures acceleration signals at a 1 min interval. In specifics, this study aimed to determine the performance score of the collar-fitted triaxial accelerometer sensor with a 1 min sampling interval in classifying the active, eating, and resting cattle behaviors using nine classification algorithms and 3 min, 4 min, and 5 min window sizes (3 data/min, 4 data/min, and 5 data/min, respectively).

## 2. Materials and Methods

**Figure 1:**
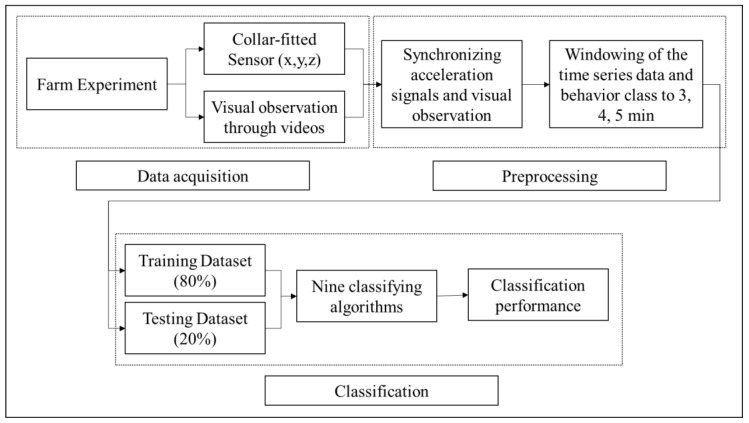
The process of evaluating the collar-fitted accelerometer sensor begins from the experiment in the field until the analysis of data and determination of the performance score of the classification algorithms used to identify behaviors of the cattle.

The flow of the methodology in evaluating the collar-fitted accelerometer sensor as shown in Figure 1 consists of data acquisition, data preprocessing, and data classification. The sub-steps of each major method were discussed further in the succeeding sections of the study.

### 2.1. Animal and Accelerometer Sensor Setup

The experiment was conducted in Soosan Dairy Farm, Cheongju City, North Chungcheong Province. The animals that were used in the study were Korean steers (Hanwoo) in their early fattening period (age: 20 months) and were fed ad libitum rice straw and supplemental concentrates (71% total digestible nutrients, 13% crude protein). Drinking water was also supplied ad libitum. To correctly identify the directions of the axes of the accelerometer during data analysis, the collar-fitted accelerometer sensor was attached to the right side of the neck of the animals. The sensors were attached more than 24 h before starting the experiment to allow the cattle to acclimatize to the sensors attached to them. The attachment of the accelerometer sensors was conducted in a manner that did not cause discomfort to the cattle while ensuring the accuracy of data acquisition. The acceleration signal data were collected for three days. While the accelerometer sensors were measuring the values, surveillance cameras were also installed for video recordings for the actual observations of the behaviors of each individual cattle during the three experimental days.

### 2.2. Collar-Fitted Accelerometer Sensor for Behavior Monitoring

**Figure 2:**
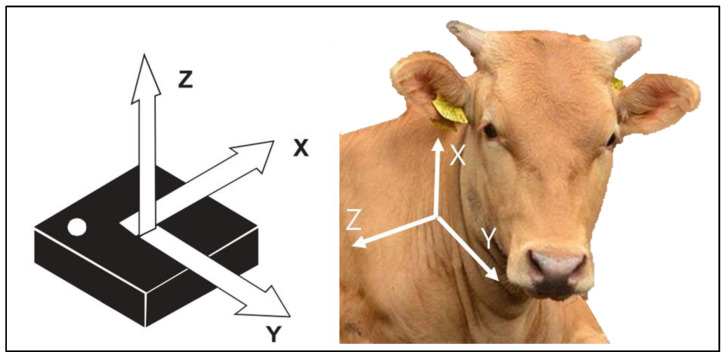
Orientation of the sensor in the Korean steer. The left figure showed the directions of the detectable accelerations on top view. The figure on the right shows the directions of the detectable accelerations when the sensor is positioned on the right neck side of the cattle. The *x*-axis measured the vertical movements of the head of the cattle, the *y*-axis measured the horizontal movements of the head of the cattle, and the *z*-axis measured the lateral, sideways, or rotational movements of the head of the cattle.

The commercial JFsystems collar device (Gunsan, Korea) consisted of a proprietary 3D LSM9DS1 accelerometer sensor positioned on the right side of the neck of the cattle to effectively detect and measure the motion patterns in the 3-axis linear acceleration sensor. The measured acceleration signals amounting to the activity of the cattle were transmitted at a 10 Hz sampling rate at a linear acceleration scale of ±2 g. The accelerometer sensor was powered with four 1.5 V alkaline batteries (750 mAh; triple A-type) and was fitted in a collar-pendant box with a dimension of 137 × 77 × 20 mm. The orientation of the sensor and directions of the 3-axis accelerometers are shown in Figure 2. The *x*-axis detected the signals in the down and up direction, the *y*-axis detected the signals in the forward and backward directions, and the *z*-axis detected the signals in lateral or sideways directions.

### 2.3. Direct Visual Observations of Behaviors through Videos

The data of the three behaviors of the cattle recorded in surveillance video cameras were observed and analyzed with trained members of the research group. The movement of the cattle was labeled per minute and manually recorded and stored in a Microsoft Excel spreadsheet. The classification and the description of the behavior of the cows (Table 1) were studied and familiarized by the observers. To ensure the accuracy of the observations, two observers tracked the video of the single cow continuously for 15 min, and thereafter, observation sessions were carried out with another member of the research group to assess and agree on the observed label of behaviors. The observation is resumed after the agreement assessment session. A label referring to the behaviors of the cows was noted every minute. As 6 h for day 1, 10 h for day 2, and 7 h for day 3 of visual observations were available for each cow, there were 360, 600, and 420 observations obtained per day, respectively. A total of 1380 visual observations were collected per individual cattle (*n* = 2). The different observation times during the three experimental days were based on the activity and inactivity of the cattle. The observation time for each day started at 7 in the morning and ended at 5 in the afternoon. The observations that were recorded were in the periods where the animals showed active, eating, and resting behaviors.

### 2.4. Combination of the Visual Observation Data and Accelerometer Signals

The data from the accelerometer sensor were wirelessly transmitted to a base station through Wi-Fi and were manually downloaded from the computer attached to the base station in a Microsoft Excel spreadsheet. At the end of the data measurements, accelerometer signals and observations were time synchronized to ensure that the visual observations were associated with the correct sequences of accelerometer signals. In this way, a dataset was obtained in which the behavior observations for each cow and each observer were combined with the appropriate sequence of accelerometer data.

### 2.5. Data Preprocessing and Preparation

The collected cattle behavior data in the X, Y, and Z axes are different, thus, the sensor orientation is a necessary condition for determining the behaviors. To accurately and smoothly reflect the motion intensity of the cattle, a summation of the acceleration signals was calculated. The collected triaxial acceleration using a collar-fitted sensor was synthesized as the following equation.

Vectorial Sum of the Acceleration (VSA) = √ ((acceleration x)2 + (acceleration y)2 + (acceleration z)2)(1)

Visual observation data of the behaviors of the animals were also relabeled into categorical numbers as follows: active was labeled as 0, eating as 1, and resting as 2. After cleaning the data of missing values, Python 3.2 [20] was used and designed to segment the VSA into long window sizes of 3 min, 4 min, and 5 min.

### 2.6. Classification Algorithm

In this study, eight commonly used classification algorithms were used for evaluating the accelerometer sensor and for comparing the three long window sizes (3 min, 4 min, and 5 min): the artificial neural network (ANN), decision tree (DT), gradient boosting (GB), k-nearest neighbor (KNN), logistic regression (LR), naïve Bayesian (NB), random forest (RF), support vector machine (SVM), and one customized classification algorithm: long short-term memory network (LSTM), a developed version of ANN for classifying time-serial-based data.

The ANN algorithm is a powerful predictive tool but needs a large amount of data to be trained to provide quality predictions. It is generally presented as a system of interconnected electronic neurons that are capable of machine learning mimicking the neural structure of the brain [21]. The algorithm process records one at a time and learns by comparing the classification of the record with the known actual classification of the record. The interconnected neuron processed the inputs which are multiplied by the synaptic weights where the product represents the strength of the neural connections. The DT algorithm is a fast, simple, and easily interpretable classification approach. As described by [22], the model is shaped like a tree with feature values in the internal nodes and class labels in the leaves of the tree, then the incoming new instances are classified by passing through the internal nodes and corresponding tests within the tree. The predicted class is the label of the final leaf reached by each instance. The GB algorithm attempts to turn a weak learner into a strong learner by gradually adapting how models are made. In order to create a strong predictive model, it combines many weak learning models together. The GB algorithm was used as it handles the noisy imbalanced data and possible outliers well [23]. The KNN algorithm is a classical non-parametric method classification algorithm that is easy to implement and suitable for multi-class classification problems used in several studies of dairy cows [18,24,25]. It is based on a neighborhood majority voting scheme and assigns the new incoming instance to the most common class amongst its K nearest. The LR algorithm is a traditional linear method utilized for predictions despite the great potential of machine learning methods because of its greater and superior fitting [26,27]. The LR classifier in the sci-kit-learn package was used to apply logistic regression to the dataset of this study. The NB algorithm is a statistical classifier based on the Bayes rule [28]. It is one of the most efficient and effective inductive learning algorithms for machine learning and data mining. This machine learning method can be used where no features before fitting the model are needed to be scaled. The RF algorithm provides a more robust classification performance by correcting overfitting to the training set [29]. The algorithm is an ensemble learning method combining multiple decision trees trained on the training set where the predictions of the individual tree models within the random forest are combined to form an overall classification decision. The SVM algorithm is a statistical machine learning method that can be applied to pattern classification and nonlinear regression tasks. The classification is performed through the non-linear mapping of input data to a higher dimensional variable space by dividing the data into two categories using linearity, and this linear separator is constructed by maximizing the distance from the training set and tuning of the SVM parameters [30]. Lastly, the architecture of the LSTM algorithm was proposed by replacing the nonlinear units in traditional recurrent neural networks with LSTM cells [31]. The common LSTM unit is composed of a cell, an input gate, an output gate, and a forget gate. The LSTM was customized based on ANN in this study to be suitable for time series data of the accelerometer signals collected using the sensors in dairy cows.

### 2.7. Performance Evaluation of the Classification

The precision, sensitivity (also called recall), F1-score, and the overall accuracy as defined by [32] were used to evaluate the classification algorithms and were calculated as follows:

Precision= TP/((TP + FP))(2)

Sensitivity= TP/((TP + FN))(3)

F1-score= (2 × Precision × Sensitivity)/(Precision + Sensitivity)(4)

Accuracy = ((TP + FN))/((TP + TN + FN + FP))(5)

where TP (true positive) is the number of positive samples correctly classified as positive samples, TN (true negative) is the number of negative samples correctly classified as negative samples, FP (false positive) is the number of negative samples incorrectly classified as positive samples, and FN (false negative) is the number of positive samples incorrectly classified as negative samples. In application, TP is the number of instances where the behavior was correctly classified using the reference observations, FN is the number of instances where the visually observed behavior was incorrectly classified, and FP is the number of instances where the behaviors were incorrectly classified according to the reference. The F1-score, in addition to precision and sensitivity, was included in this study to accurately reflect the effect of classification. The perfect effect of classification in terms of precision and sensitivity equates to 1 but the trend of these two performance parameters is often inversely related where precision increases and sensitivity decreases or vice-versa. The F1-score considers both the precision and sensitivity values by taking the weighted average of the two parameters. Hence, the closer the F1-score is to 1 is the ideal performance of the classification.

### 2.8. Statistical Analysis

To compare which of the nine (9) algorithms and three (3) window sizes showed the best classification for each behavior of the cattle, iterations of the classification algorithms were subjected to statistical analysis. For each performance variable, each of the nine algorithms per window size has ten (10) iterations of the classification, and the total number of observations was 27 (algorithms) × 10 (iterations) = 270 observations. The 270 observations for each performance variable and each behavior were subjected to the least square analysis of variance (ANOVA) using the general linear model procedure (PROC GLM) in SAS (SAS Institute, 2021). Statistically significant observations were declared at *p* < 0.05 and data were further subjected to Duncan’s multiple range test (DMRT) if differences were detected.

## 3. Results and Discussion

### 3.1. Relationship Analysis between the Acceleration Data and Behaviors of Cattle

**Figure 3:**
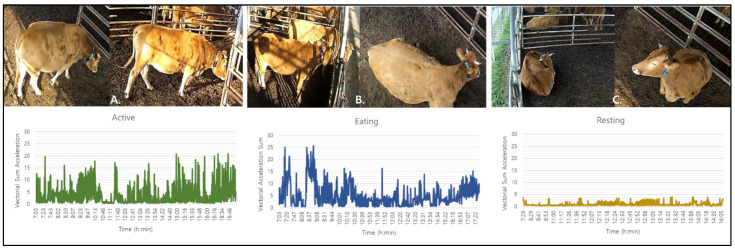
The three major cattle behaviors studied were (**A**) active behavior (standing and walking motions), (**B**) eating behavior (feeding, ruminating, and drinking motions), and (**C**) resting behavior (sleeping and lying motions). Below each behavior were the graphs of the vectorial sum acceleration per minute of the three major cattle behaviors.

The vectorial sum of the acceleration (VSA) data compared with the behaviors recorded in videos for three days is illustrated in Figure 3. The (1) active behavior characterized by standing and walking activities, (2) eating behavior characterized by feeding and ruminating activities, and (3) resting behavior characterized by lying and sleeping activities were all observed throughout the experimental period. The VSA graphs of the active and eating behaviors displayed large fluctuations showing the dynamic movements of the steers while the VSA of the resting behavior is observed as small fluctuations showing the minimal or short movements of the animals.

In Table 2, the least-square means of VSA for each behavior and the differences among the behaviors denoted by the superscripts are shown. The eating behavior showed the significantly highest mean VSA at 5.16 and the highest maximum VSA at 25.70 compared to active and resting behaviors. The notably high mean and maximum VSA values of the eating behavior were expected as the accelerometer sensor was fitted in a collar that was attached to the neck of the animals. The triaxial accelerometer detected a high activity in the neck of the cattle while the animals were eating. In a previous work [33], the feeding activity of the animals was easily distinguished from other behaviors using a neck-mounted sensor due to the high activity in the neck of the animals. This indicates that eating behavior in the present study can be highly classified compared to active and resting behaviors because of the high signals captured by the triaxial accelerometer. However, the eating behavior showed a wide range of VSA (0.40 to 25.70). The scattered VSA of the eating behavior can be associated with the erratic movement of the head of the animals as they chew and ruminate, and these dispersed signals could affect the classification of the behaviors. 

The active behavior characterized by standing and walking movements showed the second highest mean VSA at 3.76 and the second highest maximum VSA at 20.90. The standing and walking movements of the animals are normally measured by leg pedometers [34] because all movements are found in the limbs of the animals. In this study, the VSA of the active behavior measured by the collar-fitted triaxial accelerometer was high. Standing is when the body of the cattle was off the ground and supported by at least three limbs, while walking was when the cattle were moving forward or backward making two or more steps in one direction. The high VSA of the active behavior in this study, therefore, signified that the collar-fitted triaxial accelerometer sensor used in this study was effective in detecting the acceleration signals coming from the orientation of all three axes of the sensor. The triaxial accelerometer was able to sense the motion of the animals according to the orientation of the sensor with respect to the surface of the earth. 

The resting behavior characterized by the sleeping and lying activities of the animals showed the lowest mean VSA at 1.02 and the lowest maximum VSA at 4.00. Cattle are highly motivated to rest and the animals lie down for approximately 10–12 h per day [35]. When resting, the animals either lie down where their body is in the sternum position on the ground or sleep where they lie down without ruminating. Due to low movements in the lying and sleeping activities of the animals, resting was considered a durational behavior of the cattle. Thus, the low VSA signals detected by the triaxial accelerometer in the resting behavior were expected as there were little to no movements during the sleeping and lying activities of the animals. 

It was found in this study that the individual behaviors of active, eating, and resting were significantly detected by the collar-fitted triaxial accelerometer sensor. The classification performance evaluation of the sensor in classifying these individual behaviors were tested and discussed in the following section of the manuscript.

### 3.2. Precision and Sensitivity Performance Evaluation

To accurately identify the individual behaviors of cattle, nine classification algorithms were used to analyze the VSA signals measured by the collar-fitted triaxial accelerometer sensor. The performance evaluation such as the precision and sensitivity of the individual behavior using the algorithms are shown in Table 3.

The precision performance was evaluated to measure the correctly predicted positive classification over the total predicted positive observations for the individual behavior. The highest precision performance was observed in resting behavior at 89% (4 min) based on the LSTM algorithm. The second highest precision performance was observed in the eating behavior at 84% followed by the active behavior at 67% both based on a 5 min window size using the LSTM algorithm. 

The sensitivity performance was evaluated to measure the correctly positive classification over all the observations per individual behavior. The highest sensitivity performance was observed in the resting behavior at 89% in window sizes of 3 min and 5 min based on the NB algorithm. The second highest sensitivity performance was observed in the eating behavior at 88% in the 4 min window size based on the LSTM algorithm. The third highest sensitivity was observed in the active behavior at 77% in the 5 min window size based on the LR algorithm.

Among the three main behaviors of the Hanwoo steers, the resting behavior was most correctly classified in terms of precision and sensitivity performances (both at 89%) followed by eating (P: 84%, S: 88%) and then by active behavior (P: 67%, S: 77%). The high precision and sensitivity classifications of the resting and eating behaviors signified that the acceleration signals measured by the collar-fitted triaxial accelerometer sensor could not be easily misclassified as active behavior. Related works in terms of precision and sensitivity can be seen in Table 4. In comparison with other works, the precision and sensitivity scores of the resting behavior-related activities such as lying were at 83% and 80%, respectively [18]. In a recently published work, the precision and sensitivity scores of eating behavior-related activities were at 82% and 43%, respectively [14]. In older work, precision and sensitivity of feeding behaviors were 93% and 97%, respectively [36]. Lastly, the precision and sensitivity of the active-related activities such as walking were 65% and 76%, respectively [18]. Except with the work of Vasquez Diosdao on feeding behavior [36], the findings of the present study were higher if not similar to the results of the cited references. The high precision and sensitivity of the resting behavior could be due to the unscattered VSA signals measured by the triaxial accelerometer sensor. As described in the previous section of the study, the resting behavior of the cattle is composed of little to no movements of the cattle. The minimal motions in the neck of the cattle while resting could have reliably discriminated the resting behavior from the eating and active behaviors. In addition, this signified that the 1 min sampling interval of the triaxial accelerometer is reliably effective in capturing signals of behaviors that have small motions such as sleeping or lying, or collectively the resting behavior.

With eating behavior, chewing, swallowing, and under-jaw movements of the cattle could have contributed to the precise and sensitive classification of the behavior. The movements on the neck of the cattle might have contributed to the reliable discrimination of the eating behavior from resting and active behaviors. It is worthy to note that the lower precision and sensitivity scores of the eating behavior in the present study compared with the feeding behavior of cattle in the works of Vasquez Diosdao [36] could be due to the wide definition of the eating behavior in this study. The eating behavior in this study as defined in the earlier section of this study was a combination of the ruminating and feeding activities of the animals. The feeding activity includes the up and down movements of the head of the animals as well as the chewing, masticating, and other lower jaw movements while ruminating activity includes the swallowing and regurgitating activities while the animals are standing or lying. This means that the posture of the animals while ruminating could have affected the correct classificiation of the eating behavior. The lying rumination and standing rumination of the eating behavior of the Hanwoo steers could have produced proximate signals with resting and active behaviors, respectively, that caused confusion during the classification. Accelerometer sensors are tilt-sensing devices. This indicates that the triaxial accelerometer sensors used in this study were sensitive to the motions of the animals with respect to the surface of the earth. For instance, the triaxial accelerometer would measure the signals of the animals lying and the animals ruminating while lying in proximate values. Nonetheless, the results of this study indicated that eating behavior was reasonably classified using the signals measured by the triaxial accelerometer with a 1 min sampling interval.

The active behavior was poorly classified which could be due to the position of the accelerometer in the body of the animals. It was expected that the VSA signals collected for the active behavior would be from the y-axis which is associated with the forward and backward movement of the sensors. The motion of the animals with respect to the surface of the earth should give zero signals from the *x*-, *y*-, and *z*-axes when the cattle are standing, and zero signals from *x*- and *z*-axes and n signals from the *y*-axis when the cattle is walking. However, the active behavior had the second highest mean and maximum VSA as discussed in the earlier parts of the study. This indicated that the accelerometer had captured signals from all three axes giving dispersed signals, therefore, affecting the correct classification of the behaviors. The standing and walking movements of the active behavior might have been misclassified as feeding activity of the eating behavior where the body of the animals was also off the ground. The misclassification of the active behavior should be improved as low precision can lead to livestock farmers not acting on alerts of the sensor tools [37] while low sensitivity (true positives) could fail to alert the livestock farmers that there are abnormal behaviors that need attention (false negative).

Overall, high precision and sensitivity indicated that the classifiers showed minimal problems in correctly predicting positive cases and that not many negative cases were incorrectly classified as positive. The findings of the study signified that the collar-fitted triaxial accelerometer sensor measuring signals at a 1 min sampling interval could be used to classify resting and eating behaviors of cattle at high precision and sensitivity.

### 3.3. The F1-Score Performance Evaluation

To correctly classify the individual behaviors of the animals and appropriately present the results to livestock farmers, the F1-score performance was evaluated as presented in Table 3. Predicting false negatives in the behaviors of the animals could result in misdetection of the actual diseases of animals and having a misclassified actual positive in classifying behaviors poses a more serious disadvantage than predicting true observations. Hence, the F1-score was presented in this study. Eating and resting behaviors were classified with a fairly high F1-score while the active behavior was classified with a satisfactorily high F1-score. In specifics, resting behavior was the most correctly classified behavior with an F1-score of 85% in a 4 min window size followed by the eating behavior with an F1-score of 84% in a 5 min window size both based on the LSTM algorithm. The correct classification of the resting and eating behaviors of the animals in this study indicated a reasonably reliable detection of the health and welfare condition of animals. Studies reported that cattle were restless when affected with clinical mastitis [40] and cattle showed prolonged resting when affected with lameness [8,41]. Therefore, correct classification of the resting behavior in this study could reduce the misdetection of livestock farmers of probable lameness and mastitis in cattle. Other works reported that decreased feeding and ruminating activities occur in cattle affected with reduced reproduction [3] and metabolic disease [1,3]. Hence, correct classification of the eating behavior in this study could increase the detection of metabolic diseases and abnormal reproduction in cattle. The lowest F1-score was observed in the active behavior at 70% in a 5 min window size using the GB algorithm. The misclassification of the active behavior was most likely discriminated due to the positioning of the accelerometer sensor which measured mainly the movements of the neck and the head of the animals.

In comparison with related works as shown in Table 4, the F1-score for resting, eating, and active behaviors in this study was similar to the works of Smith [18]. As discussed in the above section, the F1-score for resting, eating, and active behaviors in the study was 85, 84, and 70, respectively. Similarly, works of Smith showed the F1-score of resting at 85%, eating at 86%, and active at 70%. In the cited reference, the authors used a one-vs-all framework where a set of binary classifiers were trained to discriminate each one of the behavior classes against all the combined remaining behaviors. In this study, the approach was to classify the behavior using a single classifier trained to discriminate between multiple behaviors. It was realized in this result that if the classification approach was developed in the manner of the one-vs-all scheme, the F1-score of the triaxial accelerometer sensor measuring a 1 min sampling interval could be improved further. In comparison with a newer work [39], the F1-score of eating behavior and active behavior in the present study was found to be lower. The F1-score for eating and active behaviors of the cited reference was 91% and 87%, respectively [39]. One of the main differences in the present study and cited reference was feature extraction for classification. In the present study, a small set of statistical features were used (first to fourth orders) while the cited work used fourteen statistical features. This could have contributed to the variance of the results. Nonetheless, it can be summarized that acceleration signals sampled at 1 min intervals resulted in reasonably reliable F1-score classifications of the resting and eating behaviors of the Hanwoo steers.

Among the nine algorithms, the highest F1-score (84% to 85%) was based on the LSTM algorithm for classifying resting and eating behaviors. The behaviors of cattle are generally detected and classified using time-series sensor tools. In this study, the triaxial accelerometer sensor produced time-series data for the classification of the behavior of the Hanwoo steers. In humans, it was reported that the most fitting classification model for sequential or time serial data in action and emotion recognition was the LSTM-recurrent neural network (RNN) algorithm [42]. In cattle, classification of behavior using time series activity data based on the developed LSTM-RNN model showed the best performance [43]. Similarly, the best F1-scores in classifying the resting and eating behaviors in this study were observed using the LSTM. This could be because after labeling the acceleration time series data according to actual behavior, the data were directly encoded into the classifier without further extraction of features before classification in LSTM. The LSTM was also developed to deal with the vanishing and exploding gradient problems [31] that normally occur in time-series data by learning the long-term dependence. In terms of the active behavior, the best F1-score using the GB algorithm (70%) could be explained by the capability of the algorithm to handle noisy imbalanced data such as the signals produced by the standing and walking movements. Noteworthy, the RF algorithm showed the second best F1-score in active behavior which could be due to the capability of ensemble methods of RF in generating high-performance classifiers by training the collected individual classifiers. Although the generated F1-score using RF was unsatisfactorily low (67%) for classifying the standing behavior, the RF was the second superior among the other algorithms as it has statistical, computation, and representation advantages over individual classifiers [44].

### 3.4. Classification Performance Evaluation According to Window Sizes

To address the problems of redundancy or repeating iterations and wasting of power consumption and computation costs, the behaviors of the cattle were classified using long window sizes (3 min, 4 min, and 5 min) in this study since the animals are less active. In human activities recognition, segmenting the data into smaller sizes was reported to improve classification accuracy but caused redundancy in analysis and a waste of resources [45]. Performance evaluation as affected by window sizes for active, eating, and resting behaviors are shown in Table 5. The highest precision score was observed in a 5 min window size in all behaviors. The highest sensitivity score was observed in the 4 min and 4 min window sizes in eating behavior. Window sizes did not affect the sensitivity performance in active and resting behaviors. The highest F1-score was also observed in a 5 min window size in the active and resting behaviors. Window sizes did not affect the F1-score of the eating behavior. Increasing the window sizes increases the information and instances for the classifying algorithms which improved the classification of behaviors correctly. For data in fractions of seconds, wide window sizes lead to a high percentage of unused sequences [46]. In the present study, the sampling interval was at 1 min, hence, increasing the window size increased the performance scores of the triaxial accelerometer sensor. Altogether, precision, sensitivity, and F1-score performances showed an increasing trend with increasing window sizes in all behaviors. The present study suggests that if acceleration signals were measured at a sampling interval of 1 min using a triaxial accelerometer sensor, classification of the three main behaviors active, eating, and resting in cattle generally improved at increasing window sizes.

### 3.5. Assessment of the Overall Classification Performance

The overall behavior classification performance of the accelerometer sensor based on nine algorithms and three window sizes were shown in Table 6. The overall precision, sensitivity, and F1-score were obtained by using the LSTM algorithm in window sizes of 4 min and 5 min. More specifically, the precision, sensitivity, and the F1-score of the overall classification of the behaviors of the cattle were 76–78%, 66%, and 67–71%, respectively. One of the reasons for the low classification performances in the present study could be the size of the datasets for machine learning obtained from a small number of animals (*n* = 2).

## 4. Conclusions

This study evaluated the collar-fitted triaxial accelerometer sensor designed to identify behaviors of cattle. The collar-fitted accelerometer sensor measuring 1 min serial signals was found effective for detecting the resting and eating behaviors of cattle, but needed improvement for detecting the active behavior of the animal. Based on the analysis of the minute acceleration signals and the visual observations, classifying the resting behavior resulted to 89% high precision, 81% high sensitivity, and 85% high F1-score through the combined scheme of a 4 min window size and an LSTM algorithm. Using the same combined scheme of 4 min window size and LSTM classification algorithm, the eating behavior was correctly identified at 79% high precision, 88% high sensitivity, and 83% high F1-score. However, the active behavior of the cattle was poorly classified with performance scores of 67% precision (using a 5 min window size and an LSTM algorithm), 77% sensitivity (using a 5 min window size and an LR algorithm), and 70% F1-score (using a 5 min window size and a GB algorithm). The high scores in classifying the active behavior were only attained using the 5 min window size suggesting that correct classification of the active behavior requires more data to establish a correct analysis. In addition, considering the marginally poor overall precision (78%), sensitivity (66%), and the F1-score (71%), further work is suggested to refine the 1 min acceleration signals by increasing the diversity of the inputs of classifiers or extracting more statistical features. Overall, the findings in this study may serve as critical references for engineering and animal science researchers involved in advancing smart agriculture in livestock animals.

## Figures and Tables

**Table 1:** Classification and description of the different behavior categories for actual observation.

Behavior	Definition
Active	Walking: The cow is moving from one location to another and walking straight forward with a normal gait. Standing: The cow is in an upright position on all four legs with its head in an upright position and without swinging its head from side to side, not walking, eating, or ruminating
Eating	Ruminating: The cow can be standing or lying and masticating regurgitated feed, swallowing masticated feed, or regurgitating feed with its head in an upright position. Feeding: The cow places its head above the feeding table and searches, masticates, or sorts the feed (silage), and can also be drinking
Resting	Sleeping: The cow is resting on the ground (not in an upright position) and not feeding or ruminating, settling down in a lying position and closing its eyes. Lying: The cow is resting on the ground (not in an upright position) and not feeding or ruminating but can still be moving its head

**Table 2:** The least-square means of the vectorial sum of the acceleration (VSA) for active, eating, and resting behaviors of the Korean steers.

Items	Active	Eating	Resting
Minimum	0.40	0.40	0.40
Maximum	20.90	25.70	4.00
Mean	3.76 ^b^	5.16 ^a^	1.02 ^c^
SEM	0.10	0.13	0.15

^a–c^ Means within the same row with different superscripts are statistically different (*p* < 0.05); SEM, standard error of means.

**Table 3:** Classification performance of the collar-fitted accelerometer sensor based on nine algorithms framed at different minute window sizes for specific the active, eating, and resting behaviors of the cattle.

Behavior	Classification Algorithm	Precision			Sensitivity			F1		
		3 m	4 m	5 m	3 m	4 m	5 m	3 m	4 m	5 m
Active	ANN	**62**	**63**	63	62	64	64	60	62	62
	DT	54	55	56	55	56	56	57	55	56
	GB	59	62	64	59	62	64	**64**	**66**	**70**
	KNN	56	59	61	66	65	67	60	61	64
	LR	50	51	51	**75**	**76**	**77**	59	60	60
	LSTM	52	61	**67**	64	49	57	58	52	61
	NB	54	56	58	41	42	43	46	47	48
	RF	57	60	63	64	66	71	60	63	67
	SVM	57	60	62	61	66	68	54	58	60
Eating	ANN	**60**	64	61	43	45	45	46	49	48
	DT	43	44	44	42	44	47	43	44	46
	GB	52	57	60	52	57	60	41	43	43
	KNN	48	52	56	40	44	46	42	47	50
	LR	28	28	27	19	19	18	22	22	22
	LSTM	58	**79**	**84**	**62**	**88**	**84**	**59**	**83**	**84**
	NB	44	49	52	19	24	27	25	32	35
	RF	49	53	63	39	41	44	42	45	49
	SVM	56	60	63	47	47	47	46	48	49
Resting	ANN	58	61	62	66	67	68	61	62	64
	DT	50	53	56	49	51	52	49	52	54
	GB	60	62	65	60	62	65	**62**	65	66
	KNN	60	59	61	47	54	57	51	55	58
	LR	30	32	33	41	41	42	35	36	37
	LSTM	**73**	**89**	**79**	36	**81**	86	48	**85**	**82**
	NB	44	46	47	**89**	**88**	**89**	58	59	61
	RF	57	61	64	56	60	65	56	60	64
	SVM	30	32	32	41	42	43	35	36	37

The highest performing scores as determined by the precision, sensitivity, and F1 were shown in bold format. In addition, data presented in bold and underlined format indicate the best performance scores per specific behavior. ANN, artificial neural network; DT, decision tree; GB, gradient boosting; KNN, k-nearest neighbor; LR, logistic regression; LSTM, long short-term memory; NB, naïve Bayesian; RF, random forest; SVM, support vector machine.

**Table 4:** Related works about classification performance using triaxial accelerometer in detecting cattle behaviors. Behavior.

	Algorithm	Precision	Sensitivity	F1-Score	References
Active (none)	-	-	-	-	2022 [14]
Eating (chewing)	XGB	82.00	43.00	56.00	
Resting (none)	-	-	-	-	
Active (steady standing)	RF	-	58.00	-	2022 [38]
Eating (ruminating)		-	89.30	-	
Resting (laying)		-	61.10	-	
Active (standing)	SCV	-	-	87.40	2018 [39]
Eating (ruminating)	SCV	-	-	91.30	
Resting (none)	-	-	-	-	
Active (walking)	SVM	65.00	76.00	70.00	2016 [18]
Eating (ruminating)	RFE	84.00	88.00	86.00	
Resting (as is)	RFE	83.00	88.00	85.00	
Active (standing)	DT	55.00	88.00	-	2015 [36]
Eating (feeding)		93.10	98.78		
Resting (lying)		98.63	77.42		
Active (standing and walking)	SVM	70.00	74.66	-	2009 [33]
Eating (ruminating and feeding)		83.50	75.00	-	
Resting (lying)		83.00	80.00	-	

XGB, extreme gradient boosting; RF, random forest; SCV, stratified cross-validation; SVM, support vector machine; RFE, random forest ensemble; DT, decision tree.

**Table 5:** The least square means of the classification performance of collar-fitted triaxial accelerometer sensor for the active, eating, and resting behaviors of cattle at different window sizes.

Behavior	Precision			Sensitivity			F1		
	3 m	4 m	5 m	3 m	4 m	5 m	3 m	4 m	5 m
Active	56 ^b^	58 ^a^	60 ^a^	61	63	65	57 ^bc^	59 ^ab^	60 ^a^
Eating	48 ^b^	51 ^ab^	53 ^a^	36 ^ab^	38 ^a^	40 ^a^	39	42	43
Resting	49 ^ab^	51 ^ab^	52 ^a^	56	59	60	51 ^ab^	53 ^ab^	55 ^a^

Means with different superscripts ^a–c^ within a row per performance group differ (*p < 0.05*).

**Table 6:** Classification performance of collar-fitted accelerometer sensor based on nine algorithms framed at different minute window sizes for specific behavior of cattle.

Classification Algorithm	Precision			Sensitivity			F1-Score		
	3 m	4 m	5 m	3 m	4 m	5 m	3 m	4 m	5 m
ANN	61	63	63	61	62	63	59	60	61
DT	52	53	54	51	53	54	51	53	54
GB	59	62	64	59	62	64	60	62	64
KNN	56	58	60	57	59	61	55	57	60
LR	42	42	42	54	55	55	45	46	46
**LSTM**	59	**78**	**76**	31	**66**	62	40	**71**	**67**
NB	51	53	55	47	48	50	44	46	48
RF	56	59	64	57	60	64	56	59	63
SVM	50	53	54	57	59	60	50	52	54

The highest performing scores as determined by the precision, sensitivity, and F1-score were shown in bold format. In addition, data presented in bold and underlined format indicate the best performance scores per specific behavior. ANN, artificial neural network; DT, decision tree; GB, gradient boosting; KNN, k-nearest neighbor; LR, logistic regression; LSTM, long short-term memory; NB, naïve Bayesian; RF, random forest; SVM, support vector machine.

## Data Availability

Upon a reasonable request, the data results of this study can be available from the corresponding authors.

## Abbreviations

ANN	artificial neural network
DT	decision tree
GB	gradient boosting
KNN	k-nearest neighbor
LR	logistic regression
LSTM	long short-term memory
NB	naïve Bayesian
RF	random forest
SVM	support vector machine
